# Imaging of Tonsilloliths: A Comparative Analysis of Representation Across Different Radiological Modalities

**DOI:** 10.1155/crid/6877625

**Published:** 2026-01-24

**Authors:** Adib Al-Haj Husain, Fabian N. Necker, Bernd Stadlinger

**Affiliations:** ^1^ Clinic of Cranio-Maxillofacial and Oral Surgery, Center of Dental Medicine, University of Zurich, Zurich, Switzerland, uzh.ch; ^2^ Department of Cranio-Maxillofacial and Oral Surgery, University Hospital Zurich, University of Zurich, Zurich, Switzerland, uzh.ch; ^3^ Department of Neuroradiology, Clinical Neuroscience Center, University Hospital Zurich, University of Zurich, Zurich, Switzerland, uzh.ch; ^4^ Department of Cranio-Maxillofacial Surgery, GROW School for Oncology and Reproduction, Maastricht University Medical Centre, Maastricht, the Netherlands, mumc.nl; ^5^ Incubator for Medical Mixed Reality at Stanford (IMMERS), Department of Radiology, Stanford University, Palo Alto, California, USA, stanford.edu; ^6^ Institute of Functional and Clinical Anatomy, Faculty of Medicine, Friedrich-Alexander-Universität Erlangen-Nürnberg (FAU), Erlangen, Germany, fau.de

**Keywords:** cinematic rendering, cone-beam computed tomography, magnetic resonance imaging, orthopantomogram, tonsilloliths

## Abstract

Tonsilloliths are calcifications commonly formed within the palatine tonsils, with a prevalence ranging from 10% to 40%. While typically asymptomatic, tonsilloliths can occasionally lead to complications. This case report presents a 50‐year‐old male with incidental bilateral radiopacity in the mandibular angle region detected on an orthopantomogram with no clinical symptoms. Advanced imaging modalities, including 3‐T magnetic resonance imaging (MRI), cone‐beam computed tomography (CBCT), and MR‐based cinematic rendering (CR), were employed for differential diagnosis of the observed radiopacity and preprocedural planning of extractions and implant placement in the context of the patient′s dental pathology. The consistent visualization of tonsilloliths across various imaging modalities highlights the unique strengths of each technique in diagnosing, visualizing, and differentiating these calcifications. This multimodal approach can provide valuable insights for accurate diagnosis, effective surgical planning, and improved diagnostic confidence, even in asymptomatic patients with incidental findings.

## 1. Introduction

Tonsilloliths, also known as tonsil stones, are calcifications that commonly form within the palatine tonsils, with a reported prevalence ranging from 10% to 40%, depending on the imaging modality used [1, 2]. These formations are thought to arise from the accumulation of food debris, bacteria, and cellular detritus within the crypts of the tonsils. However, despite their morphological and microbiological similarities to dental biofilm, the precise etiology of tonsilloliths remains poorly understood [3]. Tonsilloliths can present as solitary or multiple calcifications, may occur unilaterally or bilaterally, and typically measure only a few millimeters in size. In most cases, the presence of tonsilloliths is not considered pathological [4, 5]. Tonsilloliths exhibit radiopacity influenced by their mineral composition, enabling detection in approximately 50% of cases using cone‐beam computed tomography (CBCT). Notably, more than half of the tonsilloliths visualized on CBCT are also identifiable on orthopantomograms (OPGs) [6]. However, distinguishing tonsilloliths from foreign bodies or pathological calcifications in arteries, veins, lymph nodes, or salivary glands may present diagnostic challenges in conventional OPGs [7]. The primary symptom associated with tonsilloliths is halitosis, while larger calcifications may occasionally lead to additional complaints such as a foreign body sensation, sore throat, dysphagia, or cough [8]. Despite these potential symptoms, only a minority of patients report significant clinical discomfort. Treatment options vary widely and may include no intervention, mechanical removal using curettage, or, in rare cases, tonsillectomy [9].

The objective of this case report is to illustrate the complementary role of advanced imaging techniques, including magnetic resonance imaging (MRI) and MR‐based cinematic rendering (CR) reconstruction, in evaluating large palatal tonsilloliths, particularly in differentiating them from other calcified or soft tissue lesions.

## 2. Case Report

We report the case of a 50‐year‐old male patient with a history of 30 pack‐years of smoking who was referred to our clinic following incidental detection of bilateral radiopacity in the mandibular angle region on an OPG (Figure 1). The imaging sequence in this case was guided by clinical reasoning: The OPG first identified the incidental radiopacities; MRI was then performed to assess for possible soft tissue or vascular pathology; CBCT was obtained for high‐resolution three‐dimensional assessment of the calcifications and dental structures; finally, MR‐based CR was used for enhanced anatomical visualization to support surgical planning.

Figure 1(a) The present panoramic radiograph provides an overview of the dentomaxillofacial complex, revealing multiple opacities that are detectable bilaterally in the area of the mandibular ramus, adjacent to the mandibular angles. These opacities present as dense, calcified formations and exhibit an asymmetrical positioning on both sides (arrows). The morphology and density of these structures are consistent with typical calcifications associated with tonsilloliths, resulting from deposits in the crypts. Magnifications of the panoramic radiograph are shown in (b) and (c), focusing on the multifocal radiopaque structures (circle).

**Figure:**
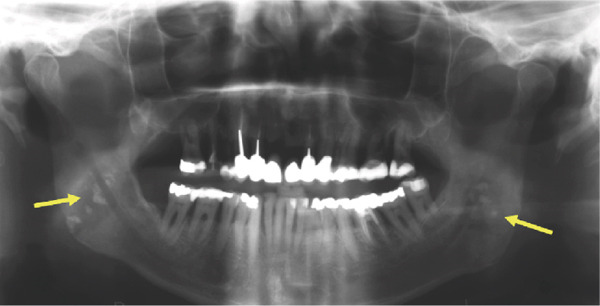
(a)

**Figure:**
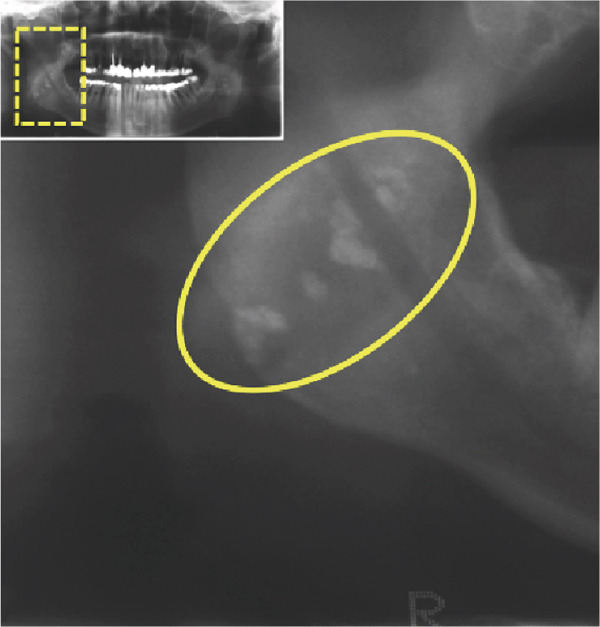
(b)

**Figure:**
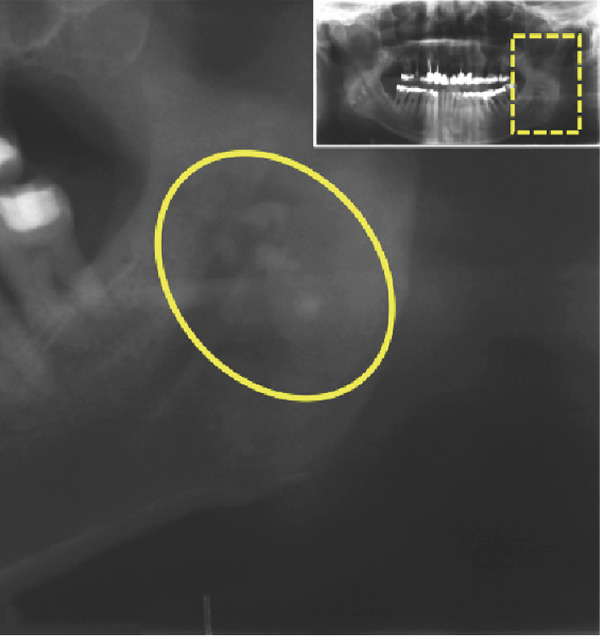
(c)

The patient was asymptomatic, and clinical examination revealed no significant findings either extraorally or intraorally. Differentiating tonsilloliths from other types of calcifications can be challenging, particularly when they involve vascular, lymphatic, or glandular tissues.

Thus, further imaging was performed to clarify these radiopaque structures, particularly considering potential atherosclerotic changes. A dental MRI scan at 3 T (MAGNETOM Skyra, release VE11E, Siemens Healthineers, Erlangen, Germany) identified corresponding focal hypointense lesions within the palatine tonsils on both T1‐ and T2‐weighted sequences (Figures 2 and 3).

Figure 2Dental magnetic resonance imaging (MRI) reconstructions ((a, b) axial; (c, d) sagittal) using the T1‐weighted, fat‐saturated Dixon gradient echo sequence (VIBE Dixon) in opposed‐phase mode reveal focal hypointense lesions in the palatine tonsils bilaterally (circle) relative to muscle tissue. Notably, there are no signs of inflammatory processes, and the surrounding soft tissues, muscles, and bone structures appear unremarkable. Additionally, no signal loss is observed in the opposed phase that would suggest pathological fat distribution or infiltration.

**Figure:**
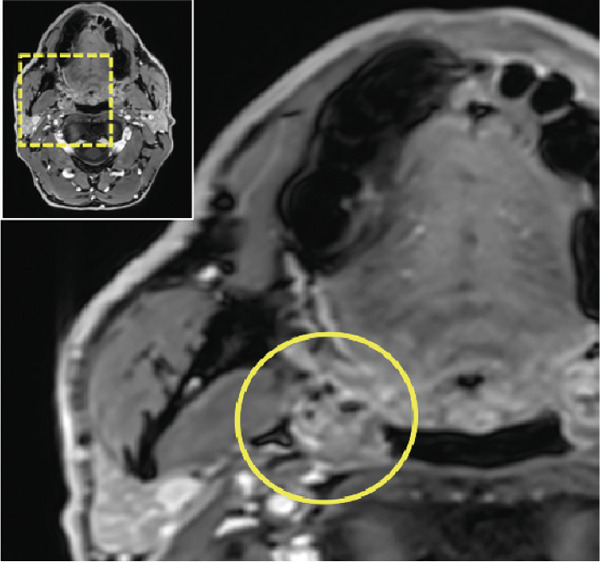
(a)

**Figure:**
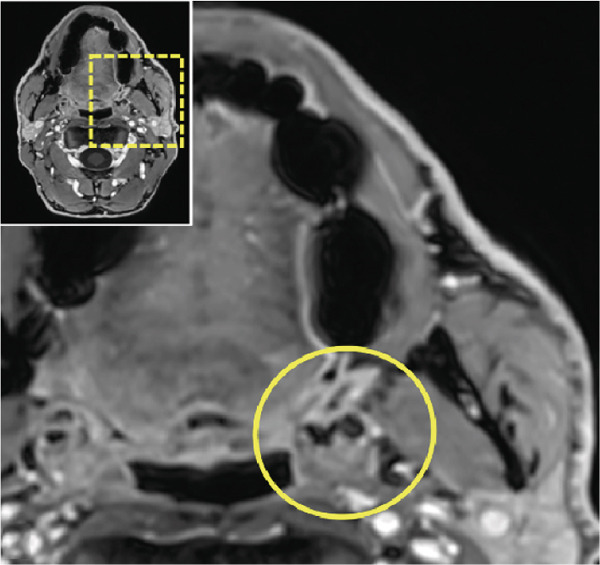
(b)

**Figure:**
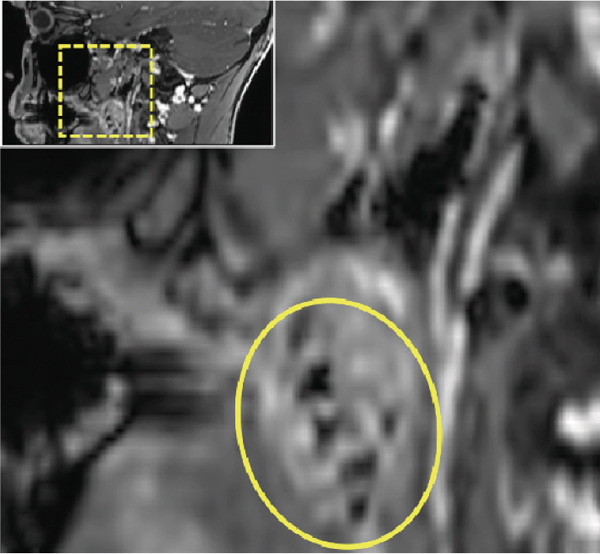
(c)

**Figure:**
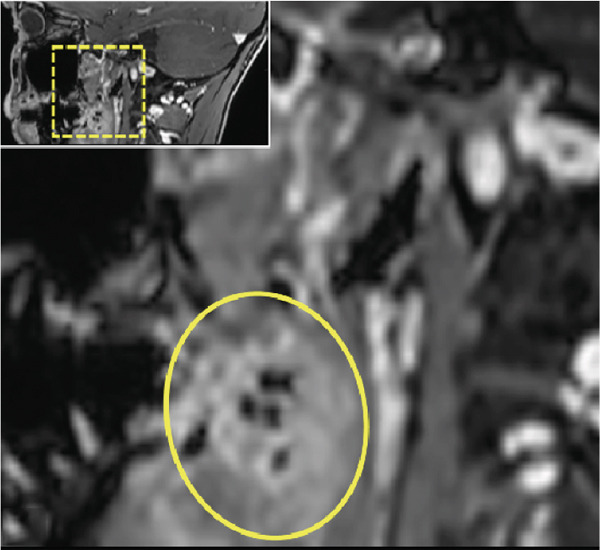
(d)

Figure 3Dental magnetic resonance imaging (MRI) reconstructions using a T2‐weighted turbo spin‐echo (TSE) sequence in the sagittal plane (a, b) reveal focal hypointense lesions in the palatine tonsils bilaterally (arrows), appearing hypointense relative to the surrounding muscle tissue. Notably, there is no evidence of inflammatory processes. The adjacent soft tissues, muscles, and bone structures appear unremarkable.

**Figure:**
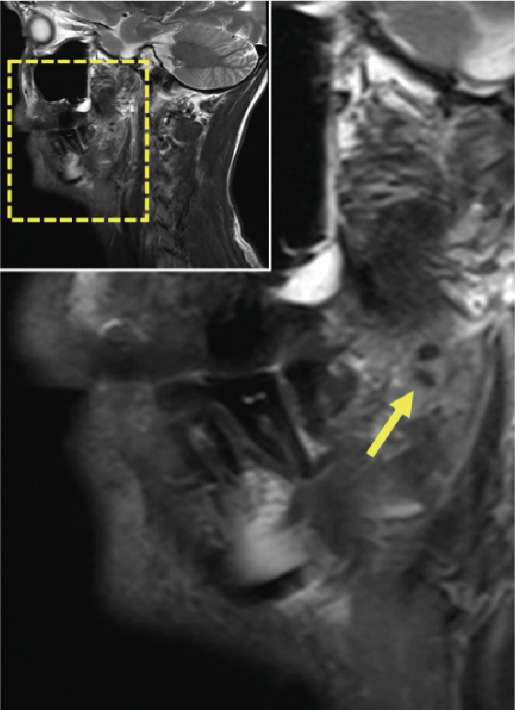
(a)

**Figure:**
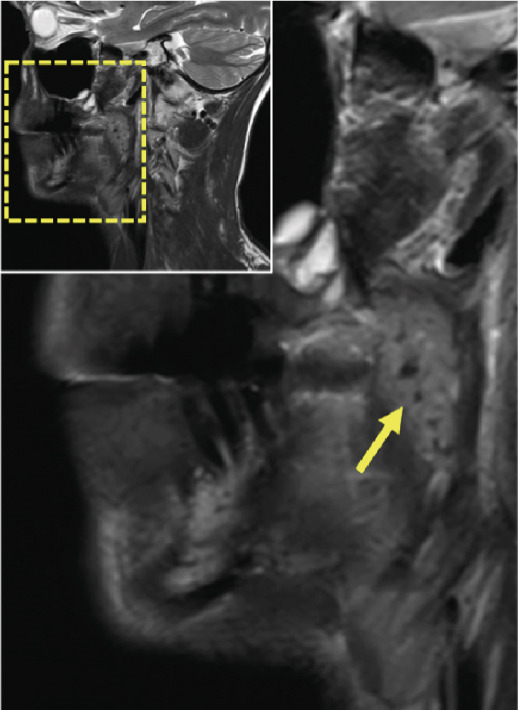
(b)

A CBCT scan (Orthophos SL 3D scanner, Dentsply‐Sirona, Bensheim, Germany) was also performed to evaluate indications for preprocedural planning of extractions and implant placement in the context of dental pathology. The scan also revealed multiple well‐defined hyperdense opacities within the palatine tonsils (Figure 4).

Figure 4The cone‐beam computed tomography (CBCT) images include (a) axial overview, (b, c) axial enlargements, (d) coronal reconstructions, and (e, f) sagittal reconstructions. In the region of the palatine tonsils, multiple well‐defined hyperdense opacities (arrows) are observed, displaying an oval to irregular shape. The dense structure of these calcifications is consistent with tonsilloliths. High‐resolution CBCT imaging provides clear visualization of the calcifications, with no evidence of surrounding soft tissue involvement or bony infiltration. The temporomandibular joint and mandible appear unremarkable.

**Figure:**
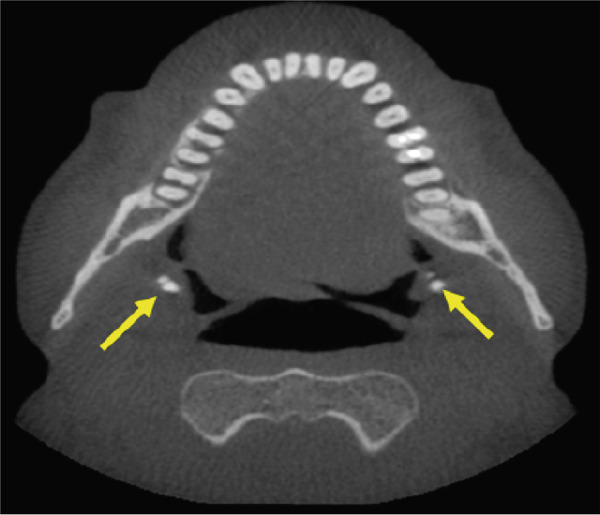
(a)

**Figure:**
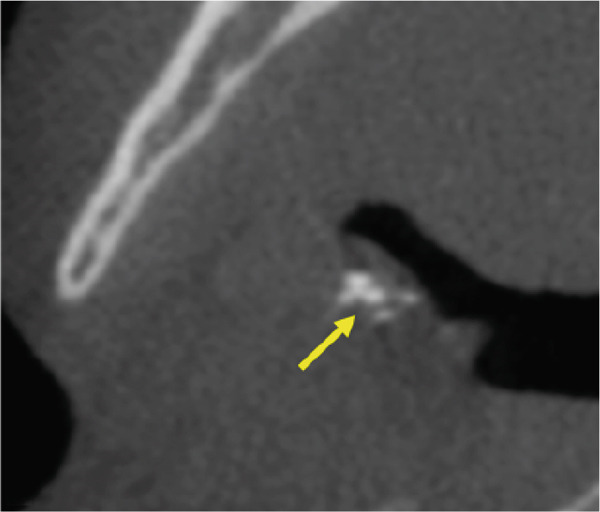
(b)

**Figure:**
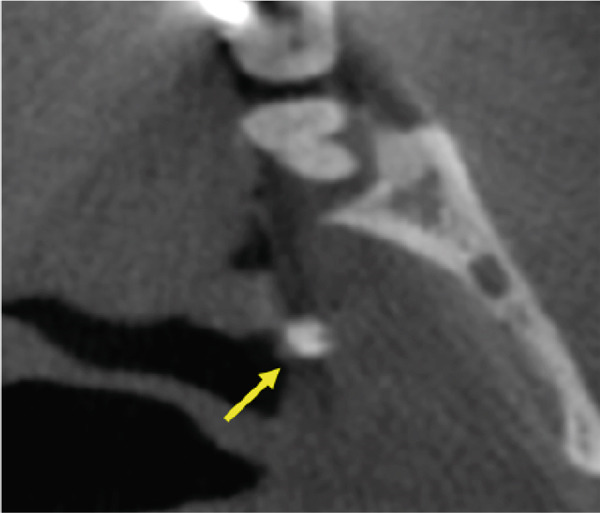
(c)

**Figure:**
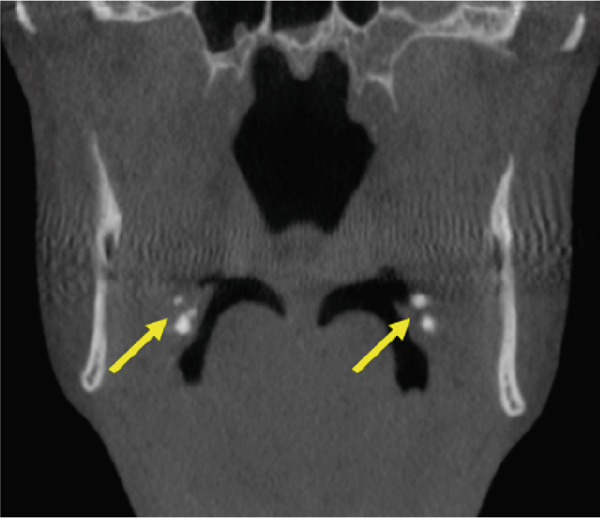
(d)

**Figure:**
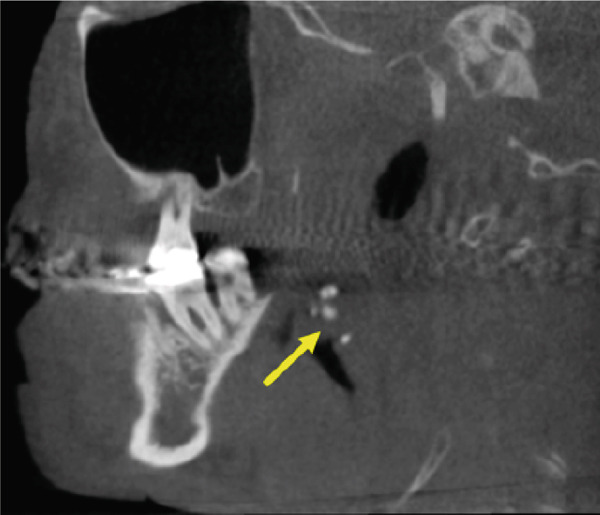
(e)

**Figure:**
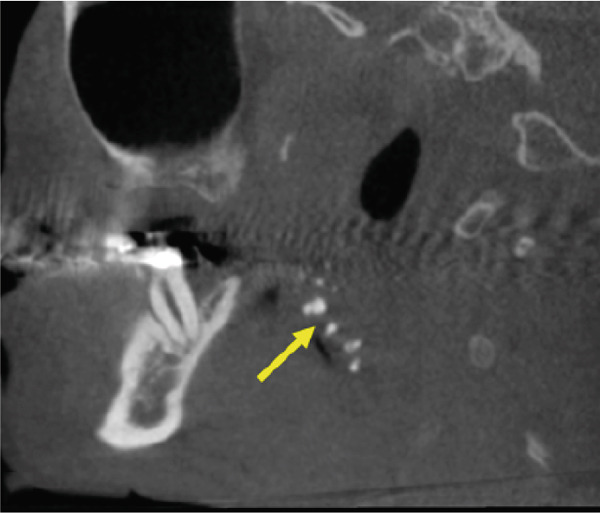
(f)

Furthermore, MR‐based CR reconstructions (Cinematic Anatomy software, CA VB128, Siemens Healthineers AG, Erlangen, Germany) were utilized to provide enhanced visualization of anatomical relationships and surrounding tissue structures (Figures 5 and 6).

Figure 5Cinematic rendering reconstructions ((a) coronal view; (b, c) lateral view magnifications) of the patient′s MR dataset clearly reveal bilateral tonsilloliths (ellipses). These three‐dimensional visualizations provide an accurate representation of the anatomical location and structure of the tonsil stones, supporting improved preoperative treatment planning. The detailed insights into the surrounding tissue conditions offered by these renderings facilitate more precise identification of potential complications.

**Figure:**
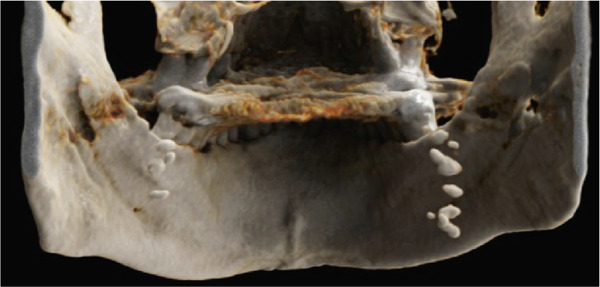
(a)

**Figure:**
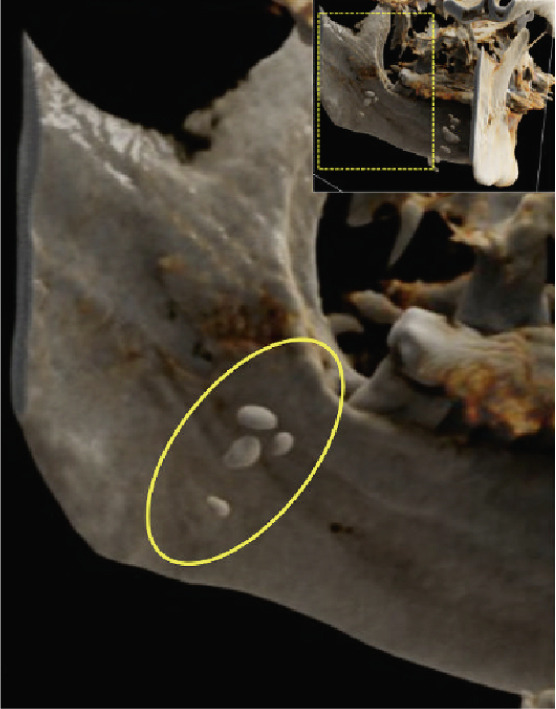
(b)

**Figure:**
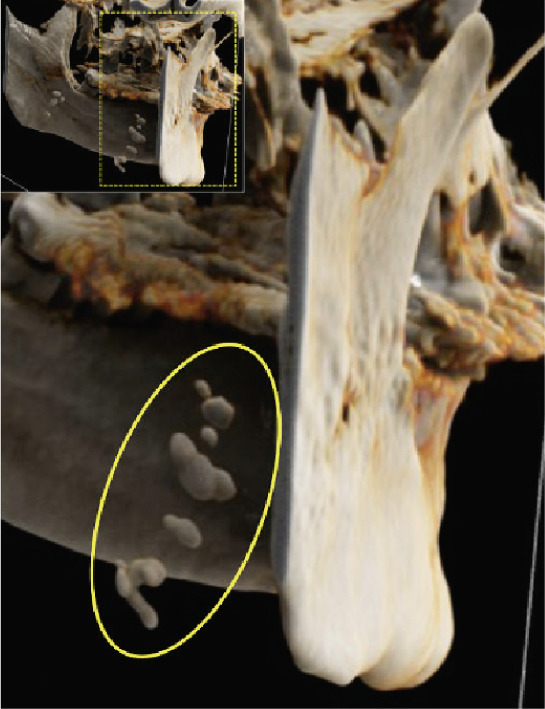
(c)

Figure 6Cinematic rendering reconstructions from the patient′s MR dataset visualize the structure of the bilateral tonsilloliths (circles) with their exact anatomical location using different presets ((a, b) soft tissue and (e, f) skin (c, d) presets), allowing for improved diagnostics.

**Figure:**
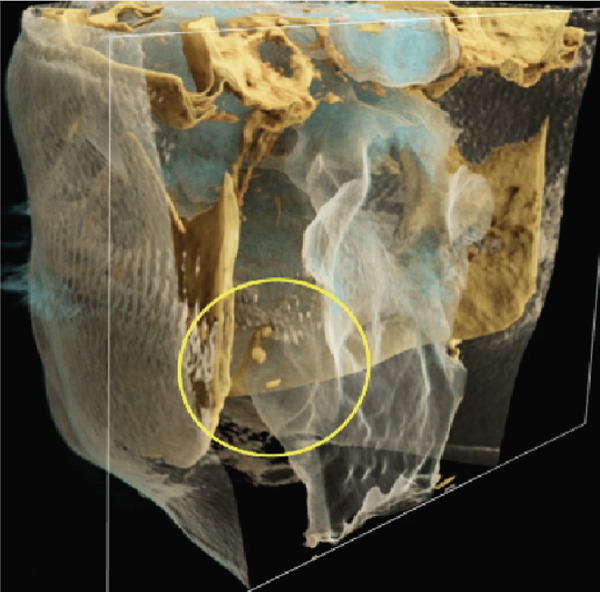
(a)

**Figure:**
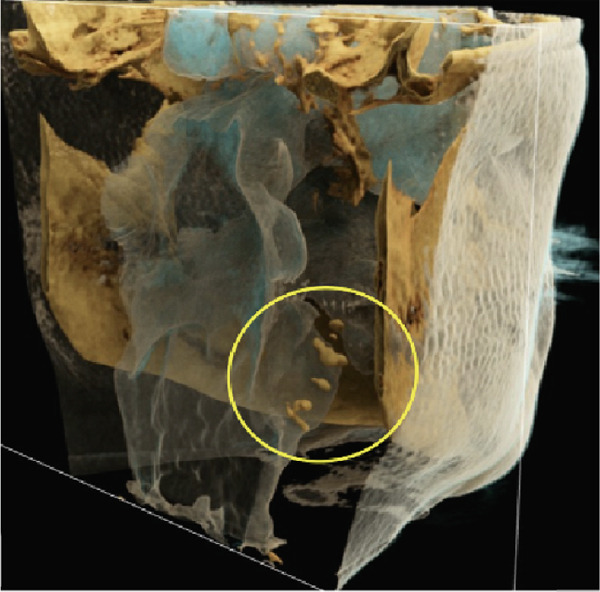
(b)

**Figure:**
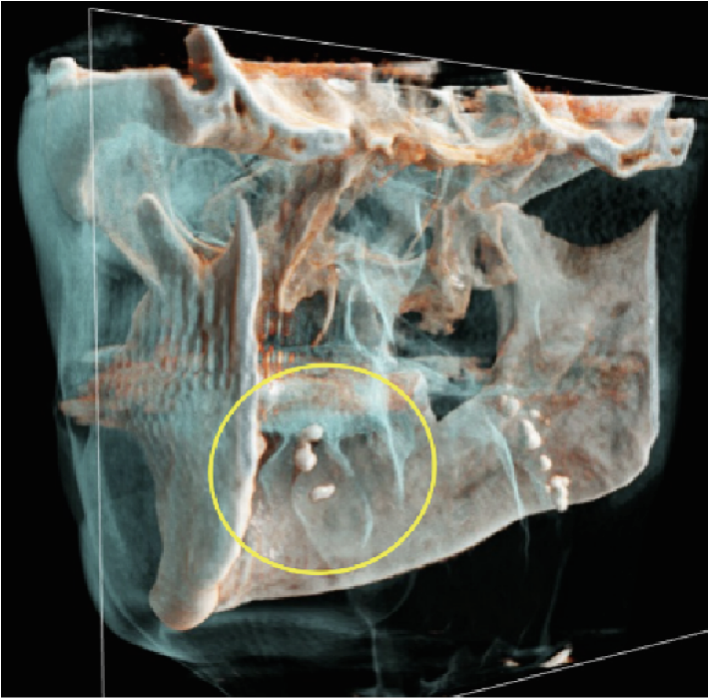
(c)

**Figure:**
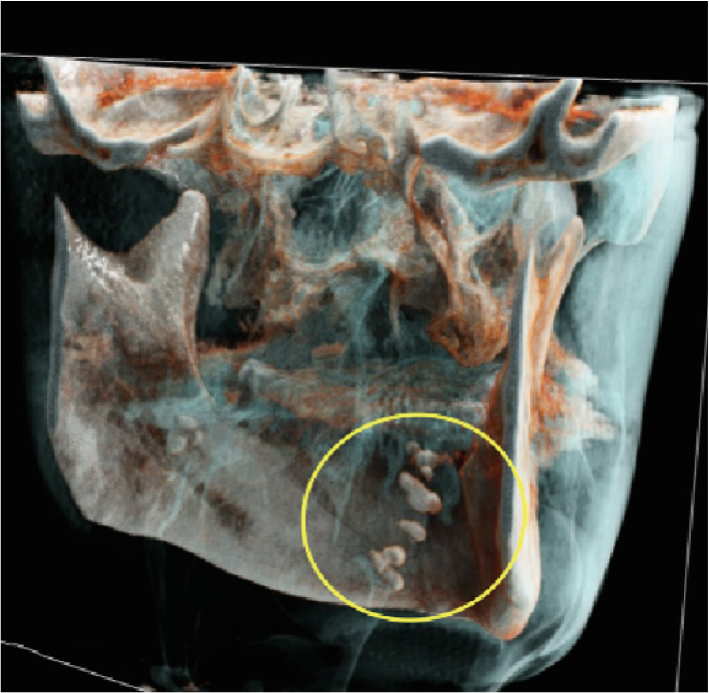
(d)

**Figure:**
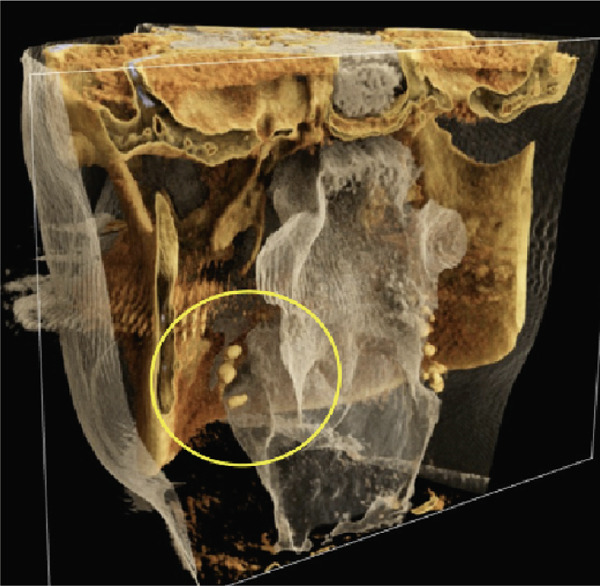
(e)

**Figure:**
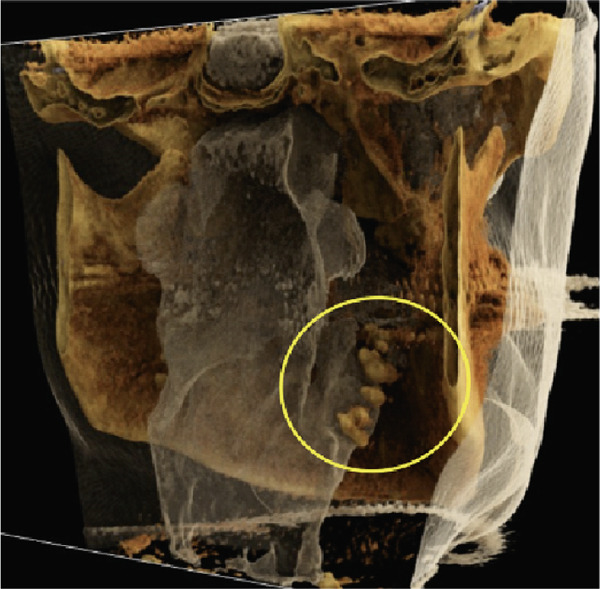
(f)

**Figure 7:**
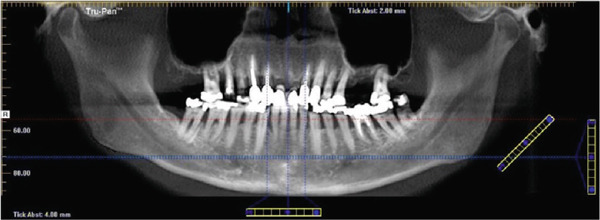
The panoramic radiograph was utilized to assess the present status of the dental pathologies and to document the surgical removal of the tonsil stones, as requested by the patient. A comparison with prior images reveals the absence of focal radiolucent structures in the region of the ascending branch of the mandibular ramus, thereby indicating the complete removal of the tonsil stones. The surgical site appears unremarkable, with no inflammation or other postoperative changes. The surrounding jaw structures remain intact, with no abnormalities detected. This image documents the successful procedure, confirming postoperative healing and the absence of recurrence.

No further therapeutic measures were recommended due to the absence of symptoms related to the tonsilloliths. Various dental treatments were performed, and an OPG was obtained to monitor the completed treatments and to plan further procedures. In the meantime, however, elective surgical removal of the tonsilloliths was performed by an otorhinolaryngologist at the patient′s request (Figure 7). Postoperative follow‐up was uneventful, with no complications or recurrence noted.

## 3. Discussion

Tonsilloliths are frequently identified in routine dental and ENT practice, and their diagnosis is often straightforward with 2D imaging and clinical evaluation. Conventional radiographic techniques, including panoramic radiography, CBCT, and CT, offer high sensitivity in detecting calcified structures. However, MRI is typically not the imaging modality of choice due to its limited ability to detect calcifications [10]. In this case, the imaging sequence was guided by clinical reasoning: The OPG first identified the incidental radiopacities; MRI was then performed to assess for possible soft tissue or vascular pathology, including atherosclerosis, phleboliths, or oropharyngeal neoplasms; CBCT was obtained for high‐resolution three‐dimensional assessment of the calcifications and dental structures; finally, MR‐based CR was used for enhanced anatomical visualization to support surgical planning.

Although complications from tonsilloliths are rare, they can lead to significant clinical outcomes, such as peritonsillar abscesses, if a stone breaches the tonsillar capsule. In rare cases, aspirated tonsilloliths have caused pulmonary complications, particularly in older patients. A history of recurrent throat infections is often associated with tonsilloliths, but most patients remain asymptomatic, with the stones frequently identified incidentally on OPGs as singular or multiple well‐defined radiopaque structures [8].

Radiographically, one key differential diagnosis in the imaging evaluation of tonsilloliths is vascular calcification, particularly atherosclerosis, which can present as radiopaque foci in the tonsillar region on OPG and CBCT images [11]. Unlike tonsilloliths, atherosclerotic plaques are typically found along the course of the carotid arteries and may exhibit a distinct distribution pattern on imaging. In contrast, phleboliths, often associated with venous malformations, typically exhibit a laminated or concentric ring‐like appearance [8]. In the present case, MRI ruled out vascular lesions by confirming the absence of signal alterations along the carotid arteries or within adjacent soft tissues, and CBCT confirmed that the calcifications were confined to the palatine tonsils. Although MRI is not typically the first‐line modality for detecting calcifications due to its lower sensitivity to highly mineralized structures [12], it was selected in this case to assess possible soft tissue involvement and to exclude pathologies that could mimic tonsilloliths but involve significant vascular or neoplastic features. By confirming the absence of imaging evidence for additional pathologies on MRI, the differential diagnosis could be refined and support the decision for conservative management. Additionally, the consistent visualization of tonsilloliths on various imaging modalities in the presented case highlights the distinct strengths of each modality in the diagnosis, visualization, and differentiation of tonsilloliths. OPGs offer an initial overview of calcifications, while CBCT provides high‐resolution, three‐dimensional data for precise localization. MRI can further complement these findings by offering superior soft tissue contrast. The cinematically rendered image was reconstructed from a conventional, clinically acquired MR imaging dataset in under 2 min [13], providing an intuitive understanding of spatial relationships that may assist in both diagnosis and surgical planning in clinical routine [14–16].

These multimodal insights are especially valuable for dentists in interpreting incidental findings, such as carotid artery calcifications, tonsilloliths, and calcified lymph nodes, providing a more comprehensive understanding of the patient′s condition. These structures can project onto various jaw regions, making them challenging to interpret on two‐dimensional OPG images. Additionally, this case report highlights the importance of raising dentists′ awareness about screening for atherosclerosis and other cardiovascular conditions, thereby enhancing routine diagnostic imaging and facilitating early intervention [17].

A limitation of this report is that it describes a single asymptomatic patient, which precludes drawing general conclusions. The imaging protocols and choice of modalities may not be applicable to all cases, particularly in patients with varying clinical presentations. Therefore, further cross‐modality comparative studies involving larger patient groups are required to explore the depiction of these pathologies.

In conclusion, while history, clinical assessment, and x‐ray‐based imaging techniques, such as OPG and CBCT, remain the preferred imaging modalities for assessing tonsilloliths in clinical practice, MRI can provide complementary diagnostic information in selected cases where soft tissue differentiation is necessary. The indication‐specific use of advanced imaging techniques not only enhances diagnostic accuracy for tonsilloliths but also highlights the complementary roles of each modality in providing comprehensive patient care in modern dentistry and otorhinolaryngology.

## Ethics Statement

All procedures followed were in accordance with the ethical standards of the responsible committee on human experimentation (institutional and national) and with the Helsinki Declaration of 1975, as revised in 2008. Informed consent was obtained from all patients for being included in the study.

## Consent

Written informed consent for publication was obtained from all participating patients involved in the study to publish research findings.

## Disclosure

All authors read and approved the final version of the manuscript.

## Conflicts of Interest

The authors declare no conflicts of interest.

## Author Contributions

All authors contributed to the study conception and design. Data acquisition, analysis, and interpretation were performed by Adib Al‐Haj Husain, Fabian N. Necker, and Bernd Stadlinger. The first draft of the manuscript was written by Adib Al‐Haj Husain, and all authors commented on previous versions of the manuscript.

## Funding

Open access publishing is facilitated by Universitat Zurich, as part of the Wiley–Universitat Zurich agreement via the Consortium of Swiss Academic Libraries.

## Data Availability

The datasets generated during and/or analyzed during the current study are available from the corresponding author upon reasonable request.
